# β-Sitosterol Loaded Nanostructured Lipid Carrier: Physical and Oxidative Stability, In Vitro Simulated Digestion and Hypocholesterolemic Activity

**DOI:** 10.3390/pharmaceutics12040386

**Published:** 2020-04-22

**Authors:** Yasamin Soleimanian, Sayed Amir Hossein Goli, Jaleh Varshosaz, Lorenzo Di Cesare Mannelli, Carla Ghelardini, Marzia Cirri, Francesca Maestrelli

**Affiliations:** 1 Department of Food Science and Technology, College of Agriculture, Isfahan University of Technology, Isfahan 84156-83111, Iran; y.soleimanian@gmail.com (Y.S.); amirgoli@cc.iut.ac.ir (S.A.H.G.); 2 Department of Chemistry, University of Florence, via Schiff 6, Sesto Fiorentino, 50019 Florence, Italy; marzia.cirri@unifi.it; 3 Department of Pharmaceutics, Faculty of Pharmacy and Novel Drug Delivery Systems Research Center, Isfahan University of Medical Sciences, Isfahan 81746-73461, Iran; varshosaz@pharm.mui.ac.ir; 4 Department of Neuroscience, Psychology, Drug Research and Child Health, University of Florence, via Schiff 6, Sesto Fiorentino, 50019 Florence, Italy; lorenzo.mannelli@unifi.it (L.D.C.M.); carla.ghelardini@unifi.it (C.G.)

**Keywords:** β-sitosterol, nanostructured lipid carrier, hypocholesterolemic activity, digestion, bioaccessibility, in vitro release

## Abstract

The objective of the present study was to explore the potential of nanostructured lipid carriers (NLCs) for improving the oral delivery of β-sitosterol, a poorly water-soluble bioactive component with hypocholesterolemic activity. Two β-sitosterol formulations with different solid lipid compositions were prepared by melt emulsification, followed by the sonication technique, and the effect of storage conditions and simulated digestion on the physical, chemical and oxidative stability, bioaccessibility and release were extensively studied. Both NLC preparations remained relatively stable during the four weeks of storage at different conditions (4, 25 and 40 °C), with more superior stability at lower temperatures. The in vitro digestion experiment indicated a high physical stability after exposure to the simulated mouth and stomach stages and an improved overall β-sitosterol bioaccessibility at the end of the digestion. The NLCs presented an increased solubility and gradual release which could be justified by the remarkable affinity of β-sitosterol to the complex lipid mixture. An in vivo study demonstrated an improved reduction in the total cholesterol and low-density lipoprotein cholesterol plasma levels in mice compared with the drug suspension. These investigations evidenced the potential of the developed NLC formulations for the enhancement of solubility and in vivo performance of β-sitosterol.

## 1. Introduction

It is well established that an increased serum cholesterol concentration is a risk factor in the development of coronary heart disease. The common treatment of hypercholesterolemia includes a class of drugs known as statins which inhibit the production of cholesterol in the liver [1]. Another way is the use of plant sterols (phytosterols) which exhibit cholesterol lowering properties due to their structural similarity to cholesterol. In comparison with routine medication prescriptions, dietary phytosterols like β-sitosterol can be considered as a financially viable strategy and when used together with hypocholesterolemic drugs, they can provide additional effects [2,3]. However, the β-sitosterol amount that people receive through diet is often not enough to provide significant blood cholesterol-lowering activity. The successful incorporation of β-sitosterol into drug preparations or functional foods is really challenging. In fact, it has a low solubility in both water and oil and it is crystalline at ambient and body temperatures. In addition, the efficiency of β-sitosterol in decreasing cholesterol is strictly dependent on the physical state and the formulation. The microcrystalline structure of β-sitosterol would increase the dose–response due to an impaired dissolution in the small intestinal fluids [4]. According to the earlier studies, consumption of 10–20 g/day of crystalline phytosterols had a comparable effect (a 10% reduction in the serum cholesterol level) with 2 g/day of solved/solubilized free phytosterols [4,5]. Similarly, Christiansen et al. (2001) found that the daily dose of 1.5 g plant sterols dispersed in spreads was enough to reach a 7.5–11.6% reduction in serum total and low-density lipoprotein cholesterol [6]. Clifton et al. (2004) demonstrated different cholesterol-lowering effects of plant sterol esters according to the food matrix. Although all phytosterol-fortified products significantly lowered cholesterol levels, the greatest reduction was seen with the low-fat milk [7]. The encapsulation of phytosterols as water dispersible formulations improves its solubility and stability and is expected to provide higher cholesterol-lowering activity as well as the great opportunity to produce a wide variety of low-fat functional foods, effective in lowering the risk of cardiovascular diseases [4,5]. Nanostructured lipid carriers (NLCs), consisting of a solid lipid matrix entrapping a liquid lipid (oil) as nanocompartments dispersed in an aqueous surfactant solution, are recognized to be very suitable for the oral delivery of poorly water-soluble nutraceuticals. An increased solubility and bioaccessibility as well as a controlled release of the bioactive compound have been described as their main benefits [8]. In NLCs, a major portion of the lipid matrix is constituted by the solid lipids, leading to an effective immobilization of the encapsulated component. On the other hand, the presence of liquid lipids distorts the formation of perfect lipid crystals, ensuring an increased drug loading and decreased drug expulsion during storage [9].

In our previous works, in order to design an appropriate formulation of a β-sitosterol NLC, a suitable lipid mixture based on functional pomegranate seed oil (PSO) and propolis wax (PW) was selected and the effect of the formulation variables (oil and drug content) and environmental conditions (thermal processing, pH, and ionic strength) on the NLC characteristics were investigated. The results indicated that this type of nano carrier could be an efficient and promising delivery system to solubilize crystalline β-sitosterol due to the broad health-promoting properties of PSO and PW, excellent compatibility of the drug and lipid matrix, high encapsulation efficiency (>90%), and the superior physical and chemical stability to a wide range of environmental stresses [9,10].

In this study, firstly the influence of storage conditions on the physical and chemical stability of the NLC was evaluated and then the ability of the developed nano carriers in improving the release and bioaccessibility of β-sitosterol was assessed using an in vitro gastrointestinal model. Finally, the in vivo pharmacodynamics of β-sitosterol entrapped in the NLC were also investigated by comparing its hypocholesterolemic activity with that of β-sitosterol in the form of a suspension.

## 2. Materials and Methods

### 2.1. Materials

The β-sitosterol was supplied by Sigma-Aldrich (St. Louis, MO, USA). The propolis sample was obtained from Espadana Mokamel Co. (Isfahan, Iran) and the propolis wax (PW, melting point of 62–64 °C) was extracted from propolis using soxhlet apparatus and petroleum ether as the solvent. Pomegranate seed oil (fatty acid composition of 78.12% ± 0.16% punicic acid, 7.6% ± 0.04% linoleic acid, 7.12% ± 0.04% oleic acid, 3% ± 0.04% palmitic acid and 2.52% ± 0.02% stearic acid) was provided by a local supplier. Compritol^®^ 888 ATO US/NF (glyceryl behenate (GB), a mixture of ~15% monoglycerides, 50% diglycerides, and 35% triglycerides of behenic acid, melting point of 71–74 °C) was kindly provided by Gattefossè (Saint-Priest, France). Lecithin (l-α-phosphatidylcholine) was purchased from Dae-Jung Co. (Shiheung, Korea). The bile extract (porcine), gastric mucin (from porcine stomach), pepsin (from porcine gastric mucosa), and lipase (from porcine pancreas, Type II) were purchased from Sigma-Aldrich Co. (St. Louis, MO, USA). All other chemicals were analytical grade and supplied by Merck Co. (Darmstadt, Germany).

### 2.2. Preparation of β-Sitosterol Loaded NLC Formulations

Two NLC formulations different in their compositions of the solid lipid phase containing 1% (*w/w*) β-sitosterol were stabilized by Tween 80 and lecithin (1:0.25 *w/w*) and produced by the melt emulsification technique, as described earlier [10]. The total concentration of the lipid phase (the mixture of solid lipids, PSO, and β-sitosterol) and surfactant mixture was 10% and 6% of the total formulation weight (20 g), respectively.

For the NLC preparation, the aqueous phase (0.96 g Tween 80 + 16.8 g phosphate buffer solution (PBS, 10 mM; pH = 7) and lipophilic phase (0.24 g lecithin + 0.2 g β-sitosterol+ 0.9 g PSO + 0.9 g solid lipid: PW alone or it’s binary mixture (1:1 *w/w*) with GB, abbreviated as PW and PW+GB NLC, respectively) were separately prepared and heated under stirring at 85 °C for 5 min. The aqueous phase was then added dropwise into the lipophilic phase under agitation (500 rpm). A coarse pre-emulsion was subjected to hot shear homogenization, using ultra-turrax (T25 basic, IKA Staufen, Germany) at 14,000 rpm for 10 min followed by an ultra-sonication treatment (Bandelin, Berlin, Germany; amplitude: 50%; power: 100W; probe: MS72) for 8 min (on for 2 s at intervals of 2 s). The obtained nano emulsion was cooled down in an ice bath for 30 min to recrystallize the lipid and form the NLC. Before the Fourier transform-infrared (FTIR) spectroscopy and evaluation of the antiradical activity, the NLC formulations were diluted (1:1 *v:v*) with PBS (pH = 7), frozen at −80 °C for 24 h, and then freeze-dried (ALPHA 2–4, Martin Christ Inc., Osterode, Germany) at −70 °C and 0.001 bars for 24 h.

### 2.3. Dynamic Light Scattering

The particle size, polydispersity index (PDI), and surface charge of the formulations were determined by photon correlation spectroscopy (PCS) using a Zetasizer (NanoSizer 3000, Malvern Instruments, Malvern, UK) at an angle of 90° in 0.01m width cells at 25.0 ± 0.1 °C. Colloidal suspensions were properly diluted with buffer solutions of the appropriate pH (depending on the study condition) in order to avoid scattering phenomena. Each sample was analyzed in triplicate.

### 2.4. Determination of β-Sitosterol Content

The β-sitosterol was extracted by saponification with ethanolic KOH and analyzed using the reverse-phase HPLC (Merck Hitachi, Darmstadt, Germany) method. The extraction process and chromatographic conditions were exactly the same as reported in our previous work [10].

### 2.5. Determination of Storage Stability of NLC Formulations

The lipid dispersions were divided into aliquots of 10 mL and stored in capped glass vials at 4, 25 and 40 °C in the dark for 30 days. The analysis of the physical, chemical, and oxidative stability as well as the antiradical activity was performed at appropriate time intervals (0, 10, 20, 30, and 40 days). All analyses were performed in triplicate.

#### 2.5.1. Physical and Chemical Stability

The physical and chemical stabilities of the formulations were monitored by measuring changes in their particle size, PDI, surface charge, and β-sitosterol content during storage. The degradation percentage of β-sitosterol in the NLC formulations was calculated by comparing the β-sitosterol content of the NLC at specific times and the initial amount of β-sitosterol added to the NLC formulation.

#### 2.5.2. Oxidative Stability by Peroxide Value (PV)

The International Dairy Foundation (IDF) standard method (74A:1991), which is described in detail by Shantha and Decker (1994), was applied to determine the lipid hydroperoxides, the primary oxidation products [11]. In order to do that, 0.6 mL of NLC colloidal suspension was vigorously vortexed three times with 3 mL of isooctane/2-propanol (3:2 *v/v*), followed by centrifugation for 5 min at 1500 rpm to extract the lipid phase. The upper layer was carefully removed and the solvent was evaporated under nitrogen gas [12]. Then, the oil samples were weighed and mixed with 3 mL of chloroform/methanol (7:3 *v/v*), followed by the addition of 15 μL of ammonium thiocyanate solution (30% *w/v*) and 15 μL of ferrous iron solution (prepared by reacting 0.132 M barium chloride and 0.144 M ferrous sulfate). The mixture was vortexed and allowed to react for 20 min in the dark at room temperature, before measuring the absorbance of the sample at 500 nm by a spectrophotometer (UV/Vis 1601 Shimadzu, Tokyo, Japan). The concentration of hydroperoxides was determined based on a standard curve of Fe^3+^ [11].

#### 2.5.3. Oxidative Stability by Thiobarbituric Acid Reactive Substance (TBARS)

The formation of the second oxidation product (malonaldehyde) was monitored by a thiobarbituric acid reactive substance (TBARS) analysis, according to the method of Qiu et al. (2015). The NLC suspensions (1.0 mL) were mixed with 2.0 mL of TBA (thiobarbituric acid) solution (prepared by mixing 15 g of trichloroacetic acid, 0.375 g of TBA, 1.76 mL of HCl 12 N, and 82.9 mL of H_2_O) in test tubes and placed in a boiling water bath for 15 min. The tubes were cooled to room temperature for 10 min and then centrifuged (1000 rpm) for 15 min. The absorbance was measured at 532 nm using a spectrophotometer (UV/Vis 1601 Shimadzu, Tokyo, Japan). The concentration of TBARS was calculated from a standard curve prepared with 1,1,3,3-tetraethoxypropane [13].

#### 2.5.4. Oxidative Stability by FTIR Spectroscopy

FTIR spectroscopy was also used as a supplementary method to analyze the oxidative stability, evidencing the qualitative presence of primary and secondary oxidation products, according to Haider et al. (2017) [14]. The samples were prepared by the pellet method with anhydrous KBr powder (1:100 lyophilized samples to KBr ratio). The spectra were obtained by FTIR (Perkin-Elmer Model 1600 apparatus, Wellesley, MA, USA) in the 4000 to 650 cm^−1^ region, using 16 scans at a resolution of 4 cm^−1^. The background was measured with a pellet holder, with a pellet of pure KBr.

#### 2.5.5. Antiradical Activity by 2,2-Diphenylpicrylhydrazyl (DPPH·)

The radical scavenging activity of the NLCs was evaluated using the DPPH stable radical. Briefly, 2 mL of a solution of DPPH in ethanol (0.1 mM) was added to 0.3 mL of NLC ethanolic solution (500 ppm) and allowed to react at room temperature. After 30 min, the absorbance values were measured at 517 nm against the blank (0.1 mM ethanolic DPPH**·** solution). The radical scavenging capacity (inhibition percentage) was expressed as a percentage of the DPPH radical elimination [12].

### 2.6. In Vitro Digestion

The initial NLC formulations were diluted five times with PBS (pH = 7) and passed through a three-step simulated in vitro gastrointestinal tract (GIT) model, including a mouth, gastric, and small intestine stage, according to Yang and McClements (2013) [15]. The samples were taken for physicochemical characterization after each stage.

Mouth stage: 20 g of the diluted original NLC (2% *w/w* lipid phase) was placed in a 250 mL flask with 20 g of simulated artificial saliva solution (SASS) prepared according to Sarkar et al. (2009) [16]. This mixture was adjusted to pH 6.8 and then shaken continuously at 37 °C for 10 min (100 rpm).

Gastric stage: A simulated gastric fluid (SGF) was prepared by placing 2 g NaCl, 7 mL HCl, and 3.2 g pepsin into a flask, adding water up to 1 L, and then adjusting the pH to 1.2. The sample from the mouth phase (20 g) was then mixed with the SGF at a 1:1 mass ratio, adjusted to pH 1.2, and incubated at 37 °C under continuous agitation at 100 rpm for 2 h.

Intestinal stage: The sample (30 g) from the simulated gastric stage was added to a clean beaker and then adjusted to pH 7 using NaOH. The mixture was then incubated for 2 h at 37 °C with a simulated small intestinal fluid (SIF) containing 2.5 mL pancreatic lipase (4.8 mg/mL), 4 mL bile extract solution (5 mg/mL), and 1 mL calcium chloride solution (750 mM). During the digestion, a pH value of 7 was maintained by adding alkali solution (NaOH) to the reaction chamber.

### 2.7. In Vitro β-Sitosterol Bioaccessibility

After the full in vitro digestion, 10 mL of the sample was centrifuged (4000 rpm) at 25 °C for 40 min. The emulsions separated into an opaque sediment phase at the bottom and a clear micelle phase at the top. Bioaccessibility was calculated by determining the β-sitosterol concentrations in the micelle phase (C _micelle_) and in the total media after digestion (C _total_) using HPLC, by the following equation [15]:

Bioaccessibility = C _micelle_/C _total_ × 100

### 2.8. In Vitro Release Study

An in vitro drug release of the β-sitosterol-loaded NLC formulations was carried out using the dialysis bag method. Before the test, dialysis membranes (cut off 14,000 Da, Sigma Aldrich, Oakville, ON, Canada) were washed and soaked overnight to remove glycerin. The bags were filled with a 5 g NLC suspension and gastric buffer (pH 1.2) at a 1:1 mass ratio and immersed in a 50 g gastric buffer for 2 h. The sink condition was provided by adding 2.5% Tween 80 to the release medium stirred at 100 rpm at 37 °C. After 2 h, the bag contents were transferred into a beaker and the pH was adjusted to 7.4 before mixing (1:1 mass ratio) with an enzyme-free intestine buffer (pH 7.4) and/or intestine buffer containing 1% lipase, under agitation in a water bath (100 rpm, 37 °C). The contents were then loaded into dialysis bags which were subsequently immersed in a 50 g intestine buffer (pH 7.4) containing 2.5% Tween 80 and incubated for 6 h at 37 °C under agitation (100 rpm). Then, the whole release medium was taken and the fresh buffer was replenished, and the experiment continued for another 16 h. The release profile of the free β-sitosterol suspension (10 mg/g β-sitosterol in water) through a dialysis bag was examined as a control. The experiments were carried out in triplicate (*n* = 3). At the end of each stage, the released β-sitosterol concentration was analyzed by HPLC using the same protocol described previously.

### 2.9. In Vivo Study

The hypocholesterolemic effect of the β-sitosterol NLC (selected formulation based on PW+GB) and plain β-sitosterol suspension was studied using a hypercholesterolemic mouse model, according to Katsarou et al. (2016) [17].

#### 2.9.1. Animals

Male mice (total number: 32, CD1, 25–30g, Envigo, Italy) were housed in stainless steel cages under controlled conditions (20–24 °C, 50–60% relative humidity, artificial 12-h light–dark cycle). Animals were housed in CeSAL (Centro Stabulazione Animali da Laboratorio, University of Florence) and used at least one week after their arrival. The accommodation was in the Department of Neuroscience, Psychology, Drug Research and Child Health (Florence, Italy), according to European standards as for experimental animals’ welfare (European ID-EL 09 BIO 03). The mice were kept at 23 ± 1 °C with a 12-h light–dark cycle, light at 7 a.m., and were allowed ad libitum access to tap water and food. All animal manipulations were carried out according to the Directive 2010/63/EU of the European Parliament and of the European Union Council (22 September 2010) on the protection of animals used for scientific purposes. The ethical policy of the University of Florence complies with the guide for the care and use of laboratory animals of the US National Institutes of Health (NIH Publication No. 85–23, revised 1996; University of Florence assurance number: A5278-01). Formal approval to conduct the experiments described was obtained from the animal subjects review board of the University of Florence. Experiments involving animals have been reported according to ARRIVE guidelines [18]. All efforts were made to minimize animal suffering and to reduce the number of animals used.

#### 2.9.2. Diet

The mice were divided into four experimental groups (*n* = 8). Group I (control group) was fed a normal laboratory diet (24% protein, 11% fat, 48% carbohydrates, 5.5% fiber, 6% vitamin 5.5% ash) and treated orally with a vehicle (1% carboxymethylcellulose); group II was fed on a high-cholesterol diet (HCD) composed of a normal diet supplemented with 2% cholesterol and treated orally with a vehicle. Groups III and IV were given a HCD and received 40 mg/kg/day β-sitosterol in the form of a suspension in 1% carboxymethylcellulose and β-sitosterol formulated as an NLC (PW+GB NLC), respectively. In all trials, oral treatments were done twice daily and continued for 60 days. Plasma samples were analyzed for total cholesterol (TC), low-density lipoprotein cholesterol (LDL), high-density lipoprotein cholesterol (HDL) fractions, and triglycerides (TG) using a Reflotron reflectance photometric analyzer (Reflotron, Roche Diagnostics and Vitros, Johnson & Johnson) and determined in mg/dL. These analyses were performed on days 30 and 60, as these are the most informative times regarding early and established metabolic damage.

### 2.10. Statistical Analysis

All measurements were carried out in triplicate and reported as means ± standard deviations. The results were analyzed using a completely randomized design with one-way ANOVA and a least significant difference (LSD) test using the SAS statistical software package version 9.4 (SAS Institute Inc., Cary, NC, USA). Differences between means were considered statistically non-significant for *p* values > 0.05.

## 3. Results and Discussion

### 3.1. Determination of Storage Stability of NLC Formulations

The NLCs exhibited a nano-sized distribution and a sufficient electrostatic stability. The mean particle diameter of the freshly prepared β-sitosterol-loaded PW and PW+GB NLCs was 96.5 ± 0.71 and 105.5 ± 0.75 nm, respectively, which is highly preferable for the targeted delivery of active compounds, since significant improvement in bioavailability is expected when the nanoparticles are in the range of 100–500 nm [19]. The difference between the particle size of the two NLC formulations could be related to the higher melting point of GB, which leads to a higher melt viscosity, and hence decreases the efficiency of the homogenization step in reducing particle size [9]. The values of the zeta potential and PDI were −26.5 ± 0.71 mv and 0.2 ± 0.03 in the PWNLC and −25.5 ± 0.28 mV and 0.21 ± 0.01 in the PW+GB NLC, respectively, which satisfies the criteria to achieve a good physical stability (zeta potential value ≥ ±20 mV and PDI ≤ 0.25) [12].

The colloidal stability, the physical integrity, and the therapeutic activity of the drug should be protected during the shelf life of nano carriers. During storage, the lipids may undergo chemical changes such as hydrolysis/oxidation or polymorphic transitions which may compromise the stability of the entrapped drug. NLCs might be more stable under low temperatures; however, NLC preparations and foods or beverages fortified with NLCs may be stored at different temperatures. Therefore, we examined the influence of time and temperature (4, 25 and 40 °C) on the physical, chemical, and oxidative stability and free radical scavenging activity of the NLC dispersions encapsulating β-sitosterol.

#### 3.1.1. Physical and Chemical Stability

**Figure 1:**
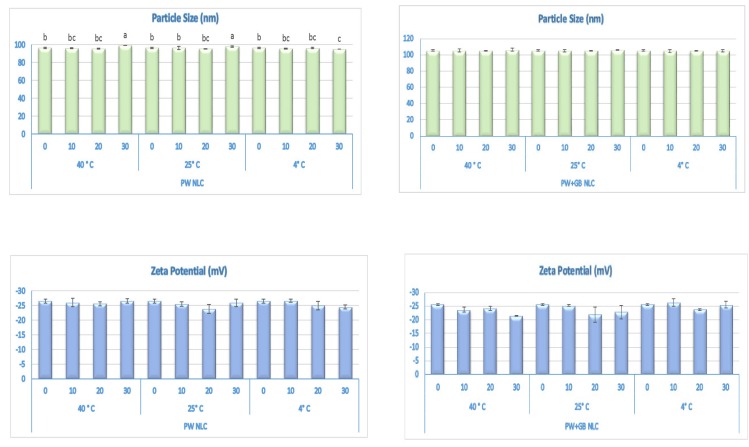

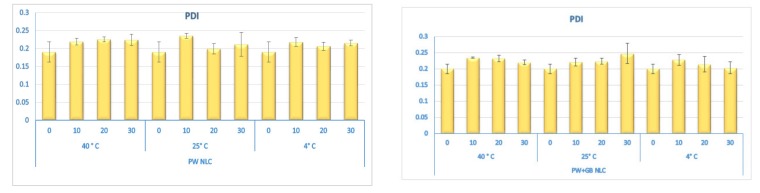
Effects of storage on the physical stability of β-sitosterol NLC dispersions at different temperatures (4, 25 and 40 °C) over time. In each parameter, the means that have no superscript in common are significantly different from each other (*p* ≤ 0.05). The means without superscript are not significantly different (*p* > 0.05). PW and PW+GB represent the NLCs formulated with propolis wax alone or its binary mixture with glyceryl behenate (1:1), respectively.

Figure 1 shows the changes in size, PDI, and zeta potential of the dispersions during storage. The visual observation of the NLCs did not indicate any sign of gelation, creaming, phase separation, or particle aggregation at the different temperatures. There was no appreciable change in the particle size and particle size distribution of the NLC formulations stored at different temperatures and an average diameter ranged between 95.5 to 99 nm in the PW and 104.5 to 106 nm in the PW+GB NLCs with a PDI < 0.25, indicating a monodisperse pattern and good quality of particle dispersion. In addition, there was hardly any change (*p* > 0.05) in the zeta potential value of the formulations throughout the storage period (Figure 1).

Previous studies have shown that the lipid matrix and nature of the colloidal interactions operating between oil droplets considerably affects the stability of lipid nanoparticles [10,11,12,13,14,15,16,17,18,19,20]. In this study, wax-based NLCs were stabilized by Tween 80 and lecithin. Wax carriers exhibit excellent physical properties and a kinetic barrier to Ostwald ripening due to the large proportion of long chain fatty acids [10]. On the other hand, surfactants like lecithin and Tween act as strong crystallization inhibitors and can ensure the long-term stability of phytosterol emulsions [4]. In addition, the small size of the droplet causes a reduction in the gravity force, and thus the Brownian motion would be sufficient to overcome gravity, flocculation, separation, or coalescence [21]. Similarly, Yang et al. (2013) did not observe any change in the appearance or mean particle diameter of the Tween 80-stabilized emulsions stored at 5 or 37 °C for one month [22]. Our observations were also in agreement with the findings of Khalid et al. (2017), who investigated the storage stability of Tween-stabilized oil in water (O/W) emulsions encapsulating 1% (*w/w*) β-sitosterol at 4 and 25 °C for a period of 30 days. No prominent change was observed in the particle size distribution of the emulsions, which was attributed to the strong hydrophilic nature of Tween 20 that could stabilize the oil–water interfaces for long periods. The remarkable stability profile of the emulsions was also attributed to the surface activity of β-sitosterol that reduces the interfacial free energy at the interface, and thus renders some degree of stability to the resulting emulsion system [23]. Furthermore, in studies performed by Chen et al. (2016) and Zychowski et al. (2018), phytosterols have been found to improve the overall stability of the oil in water emulsion over time [24,25].

**Figure 2:**
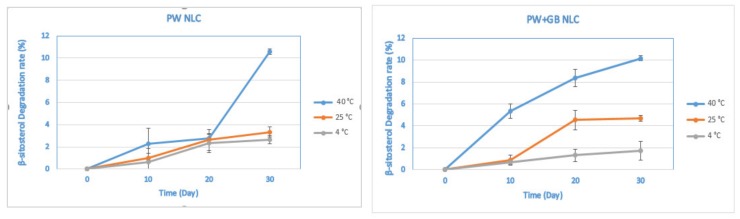
Effects of storage on the chemical stability of β-sitosterol NLC dispersions at different temperatures (4, 25 and 40 °C) over time. PW and PW+GB represent the NLCs formulated with propolis wax alone or its binary mixture with glyceryl behenate (1:1), respectively.

Figure 2 represents the chemical stability of the NLCs at different temperatures over time in terms of their β-sitosterol content. The β-sitosterol was gradually degraded during storage and by the end of the period, about 2–10% of the β-sitosterol was lost, with greater losses occurring at 40 °C than at 25 and 4 °C. At 40 °C, a significant decrease in the β-sitosterol content was observed for the PW+GB NLC on the 10th day, which was not statistically different from those reported for the next intervals (*p* > 0.05). In the case of the PW NLC, a decrease in β-sitosterol content was significant only after 30 days. In the samples stored at 4 and 25 °C, the increase in the degradation rate was not significant throughout the whole period. In comparison with the PW+GB NLC, the PW NLC samples exhibited slightly greater chemical stability; however, the difference was not significant (*p* > 0.05) on the 30th day.

Oxidation is the main known mechanism for phytosterol degradation which can give rise to a number of products including ketones, alcohols, epoxides, and dienes, depending on sterol structure, matrix composition, and reaction temperature [26]. The surface activity and physical structure of phytosterols would allow them to concentrate and pack tightly at the oil–water interface. Consequently, their sensitive hydrophilic hydroxyl groups and the double bond would be oriented towards the aqueous phase, where oxidative stress is high [26]. The stability results of our study correlated well with those obtained by Khalid et al. (2017), who reported a gradual decline (up to 15%) in the β-sitosterol content in the O/W emulsions stored at 25 and 4 °C during one-month storage.

#### 3.1.2. Oxidative Stability by Peroxide Value

Lipid oxidation is one of the most problematic deterioration processes occurring during the storage of aqueous-based nanoformulations containing unsaturated lipids, leading to the development of rancidity, off-flavors, and polymerization reactions. Lipid oxidation may also generate undesirable biologically active species that are involved in cardiovascular and inflammatory disease processes [27]. In emulsions, the interface between the oil and the aqueous phase is the place of contact between the lipids and hydrophilic pro-oxidative components (such as transition metal ions: iron and copper, photosensitizers, and enzymes). Therefore, lipid oxidation in emulsions is much faster than bulk oil, presumably due to the high surface area, and thus more interactions with pro-oxidants present in the aqueous phase [26].

**Figure 3:**
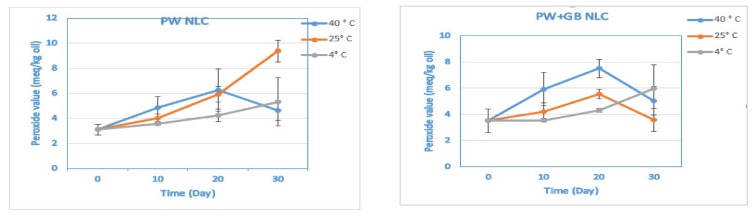
Oxidative stability of β-sitosterol NLC dispersions by peroxide value (PV) (meq/kg oil) during storage at different temperatures (4, 25 and 40 °C). PW and PW+GB represent the NLCs formulated with propolis wax alone or its binary mixture with glyceryl behenate (1:1), respectively.

The PSO used in the present study is rich in poly unsaturated fatty acids, which are highly susceptible to degradation through oxidative reactions. The extent of lipid oxidation was therefore monitored by evaluating the lipid hydroperoxides concentration during the storage trial as a marker of the early stages of oxidation. The apparent rate of hydroperoxide formation over the 30 days of storage was highest at 40 °C and lowest at 4 °C (*p* < 0.05), where there was a slight increase in the hydroperoxide concentration from 3.09 ± 0.43 (the PV of the fresh PW NLC) to 5.32 ± 1.93 meq/kg oil and from 3.51 ± 0.92 (the PV of the fresh PW+GB NLC) to 5.05 ± 1.09 meq/kg oil throughout the trial (*p* > 0.05) (Figure 3 and Table S1 in Supplementary Materials). The concentration of peroxides in all samples increased with storage time; however, some preparations such as the PW+GB NLC at 25 and 40 °C and the PW NLC at 40 °C followed a decreased trend after the 20th day of storage. It is known that hydroperoxides are the unstable primary oxidation products that are prone to breakdown. Therefore, what we observed can be a result of the decomposition of hydroperoxide to the aromatic compounds (second products of oxidation) during the later stages of oxidation. This trend of a rapid increase followed by a fast decrease in lipid hydroperoxide concentrations has been previously reported in other oil in water emulsion systems [26,28,29].

#### 3.1.3. Oxidative Stability by Thiobarbituric Acid Reactive Substance (TBARS)

**Figure 4:**
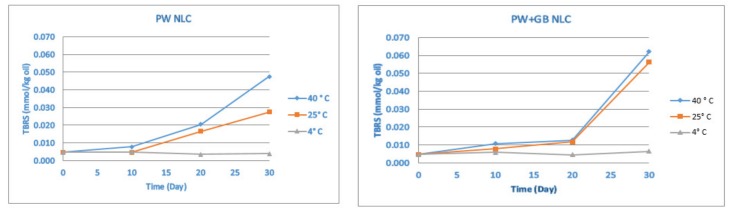
Oxidative stability of β-sitosterol NLC dispersions by thiobarbituric acid reactive substance (TBARS) (mmol/kg oil) during storage at different temperatures (4, 25 and 40 °C). PW and PW+GB represent the NLCs formulated with propolis wax alone or its binary mixture with glyceryl behenate (1:1), respectively.

The oxidative stability was further monitored by a TBARS value which evaluates the formation of the second products of the oxidation (malonaldehyde) and provides a more comprehensive view on the oxidation. There was a negligible rate of malonaldehyde formation at 4 °C throughout the trial (*p* > 0.05). In contrast, in the samples stored at 25 and 40 °C, the TBARS value remained roughly constant for about 10 days, followed by an increase and reaching of the maximum at the end of the period. According to Figure 4, the formation of the secondary products of the oxidation was significant after 20 days of storage, which correlated well with the changes observed in the peroxide value (the decomposition of hydroperoxides began after 20 days).

Similarly, in Nasrabadi et al.’s (2016) study, the PV of conjugated linoleic acid (CLA) oil in a water emulsion stored at 50 °C began to decrease and the TBA value increased significantly after 3 weeks as a result of hydroperoxide degradation [29].

Compared to the PW+GB NLC, the PW NLCs underwent a lower rate of malonaldehyde formation (*p* < 0.05) and the highest amount of the secondary oxidation product (0.062 mmol/kg oil) was found in the PW+GB sample after 30 days of storage at 40 °C. However, even with the increasing TBA value, PSO protection was still guaranteed because a significant formation of malonaldehyde in the first 20 days was prevented. Pomegranate seed oil is rich in polyphenols, including gallic acid and ellagic acid, and also tocopherols, mainly γ-tocopherol and α-tocopherol, with a high antioxidant activity, which could be the reason for the high resistance of the PSO to oxidation. Furthermore, PW is known to manifest antioxidant activity due to the presence of pentacyclic triterpenoids in its composition. Similarly, in our previous study, the NLCs (especially the PW NLC) showed very good oxidative stability after production and also during the storage, probably due to the antioxidant activity of the lipid matrix and the chemically protective effect of the solid core of the nanoparticles that prevents the oxygen from reaching the oil [12].

#### 3.1.4. Oxidative Stability by FTIR Spectroscopy

**Figure 5:**
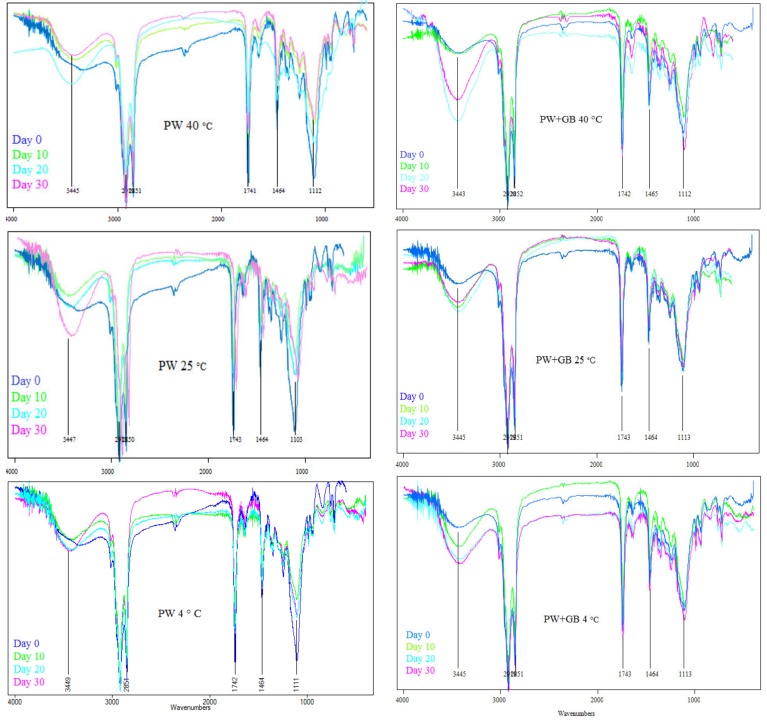
Oxidative stability of β-sitosterol NLC dispersions by Fourier Transform Infrared Spectrophotometry (FTIR) during storage at different temperatures (4, 25 and 40 °C). PW and PW+GB represent the NLCs formulated with propolis wax alone or its binary mixture with glyceryl behenate (1:1), respectively.

As mentioned in previous works, the oxidative status of an oil can be monitored by the frequency and absorbance values of the bands of the infrared spectra. Peaks in the ranges 3200–3600 cm^−1^ and 1730–1750 cm^−1^ are related to the formation of first (hydroperoxides) and secondary oxidation products (ketones and aldehydes), respectively. It was previously shown that during the oxidation process, the band in the hydroperoxides region widens and intensifies as the oxidation degree increases. However, the frequency value of this band may decrease with the progressive degradation of hydroperoxides [14,30]. Figure 5 illustrates the obvious spectral changes in the NLC spectra in the hydroperoxide region. The frequency of the maximum absorbance near 3445 cm^−1^ observed in this interval for the different samples is in agreement with other authors [31]. Both NLC preparations stored at a refrigerator temperature and the PW NLC stored at room temperature showed an increase in absorbance for this wavelength range during the trial (the band became wider and more intense as the oxidation process proceeded). On the other hand, the NLC preparations stored at 40 °C and the PW+GB NLC stored at 25 °C showed an obvious increase in intensity of the hydroperoxide band during the first 20 days of storage, which then decreased until the 30th day, suggesting the breakdown of hydroperoxides, in accordance with the results obtained by the PV and TBARS value. The same behavior was observed by Comunian et al. (2017), studying the oxidative stability of the echium oil microcapsules stored for 30 days at 40 °C [30].

#### 3.1.5. Antiradical Activity by 2,2-Diphenylpicrylhydrazyl (DPPH·)

The DPPH method has been widely used to evaluate the radical scavenging capability of various substances that transfer their labile H atoms to radicals. The PW NLC exhibited a better percentage of antiradical activity, which might be the reason for the lower oxidation rate compared with the PW+GB NLC (Table 1). Regarding the storage condition, no significant variation in antiradical activity of each formulation was observed at the different times and temperatures (*p* > 0.05).

### 3.2. In Vitro Digestion and β-Sitosterol Bio Accessibility

In the present study, we developed a simulated gastrointestinal tract (GIT) model to understand the colloidal behavior and potential biological fate of NLC systems during digestion. This knowledge helps the pharmaceutical and food industries in the rational design of emulsion-based delivery systems to encapsulate, protect, and carry functional ingredients to specific sites within the GIT and release them at a controlled rate [32].

**Figure 6:**
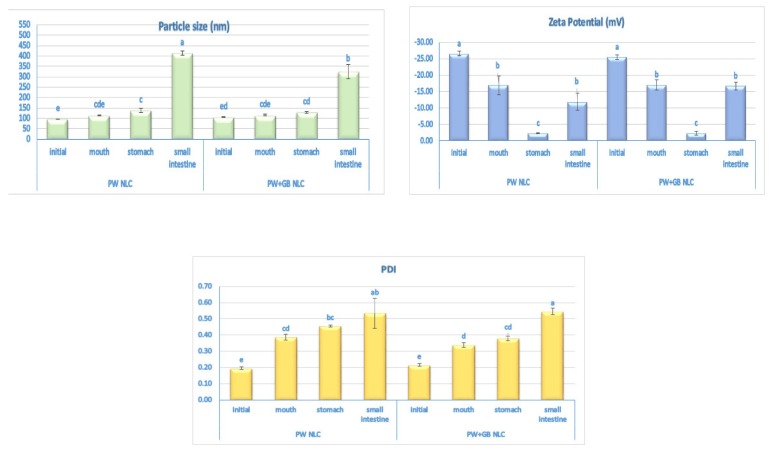
Effect of the different stages of the simulated gastrointestinal tract on the mean particle size, PDI, and surface charge of the β-sitosterol NLC dispersions. In each parameter, the means that have no superscript in common are significantly different from each other (*p* ≤ 0.05). PW and PW+GB represent the NLCs formulated with propolis wax alone or its binary mixture with glyceryl behenate (1:1), respectively.

The changes in the mean particle size and PDI of the initial NLCs collected after each stage of digestion are illustrated in Figure 6. The oil droplets were fairly stable to aggregation under the simulated mouth and gastric conditions and the mean diameter remained relatively small (<140 nm), irrespective of the carrier composition. Similarly, in our previous study, we reported little change in the mean particle diameter of the NLCs over a wide range of pH conditions (pH 2–8) [10]. This stability to a low pH and the pepsin enzyme in the gastric phase may be attributed to the strong steric stabilizing effect of Tween 80 and its insensitive nature to hydrolysis by proteases [33].

Instability and dissolution in the stomach may not be desirable if active ingredients require protection against the acidic environment, which clearly highlights the importance of interfacial technology in achieving a targeted delivery [19]. The findings were in agreement with previous studies, reporting the same behavior of Tween 80-stabilized emulsions under the simulated gastric condition [34,35,36].

In contrast, there was a remarkable reduction in the physical stability of the colloidal suspensions after exposure to the small intestine phase and the particle size increased to around 300 to 400 nm in the PW+GB NLC and PW NLC, respectively, with a PDI greater than 0.5. This instability is probably due to the alterations in the internal and interfacial composition of the oil droplets promoted by the bile salts, phospholipids and lipase of the digestion juice, formation of mixed micelles, or generation of insoluble sediments (such as calcium soaps) [15,33].

The changes in the surface charge of particles were also studied during digestion (Figure 6). After passage through the simulated oral stage, despite some reduction in the magnitude, the charge remained highly negative, which can be attributed to the anionic groups of lecithin at neutral conditions within the mouth. The changes in charge may have been a result of the adsorption of some ionized components of the simulated saliva such as minerals or mucin to the droplet surfaces [15]. In contrast, an appreciable reduction in the electrical charge of both NLC systems (to about −2 mV) was observed after incubation in the simulated gastric fluid, as a result of the protonation of the anionic groups of lecithin and ether linkage of Tween 80 at low pH values (Figure 6). However, extensive particle aggregation was prevented, suggesting that a sufficient steric stability provided by the non-ionic emulsifier can compensate the weak electrostatic repulsion [10]. Exposure to the simulated intestinal fluid caused a large increase in the magnitude of the negative charge on the droplets, which could be attributed to the negative charge of lecithin at a neutral pH and the binding of the anionic surface active substances of the digestive juices, like phospholipids and bile salts, to the particle surfaces. In addition, lipase converts neutral triacylglycerol molecules into anionic-free fatty acids which may remain at the surfaces and produce a negative charge [15].

The above results indicated that the NLCs remained almost physically stable and in the colloidal nanometer range (≤550 nm) within which digestion may increase their bioaccessibility at the small intestine. We therefore quantified the bioaccessibility by measuring the β-sitosterol concentration within the micelle phase and the total digestion fluids collected at the end of the experiment. The developed NLCs were effective in improving β-sitosterol solubility and the overall bioaccessibility was determined around 69% and 73% in the PW and PW+GB NLCs, respectively. The interaction of the wall materials with the digestive system plays an important role in solubilizing the drug. Earlier studies reported that the incorporation of highly hydrophobic drugs into the lipid carriers promotes the formation of mixed micelles and makes a significant enhancement in oral bioavailability [15,33,37,38]. The solubilization capacity of mixed micelles depends on the nature of the free fatty acids and mono-acylglycerols released after the carrier lipid digestion. In comparison with medium chain fatty acids, lipids containing long chain fatty acids may increase the overall bioaccessibility by the formation of mixed micelles with a larger hydrophobic core, and thus a higher solubilization capacity [15,33,37]. Furthermore, the bioaccessibility of lipophilic nutraceuticals increase as the rate of lipid digestion does, and therefore the total amount of mixed micelles increases [15]. Qian et al. (2012) reported the lowest β-carotene bioaccessibility in nanoemulsions containing indigestible flavor oils, such as orange oil, as the carrier lipid [37].

### 3.3. In Vitro Release Study

In vitro drug release studies also play an important role in the prediction of the formulation behavior during the various stages of digestion. Depending on the composition, different mechanisms are involved in the release process of nanoparticles, including matrix erosion (hydrolytic degradation), diffusion, and drug release from the nanoparticle surface [39]. In lipid nano carriers, drug release is mainly governed by diffusion and erosion and is expected to be slower from the more lipophilic matrices and carriers with a higher encapsulation efficiency and lower degree of crystallinity. In NLCs, the presence of the liquid lipid results in a less-ordered structure which better accommodates the drug and prevents its expulsion [39,40]. In addition to the type of carrier, the release profile may be affected by the drug’s physicochemical properties and the incorporation method of the drug into the formulation [39]. In the study carried out by Yuan et al. (2007), NLCs produced by the solvent diffusion method presented a faster drug release compared with those obtained by the melt emulsification technique [41].

**Figure 7:**
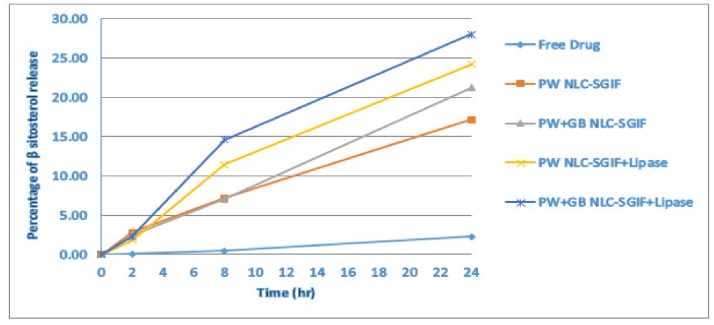
In vitro release profiles of the β-sitosterol-loaded NLCs in the simulated gastro-intestinal fluid (SGIF) with or without a lipase in comparison with a free drug suspension. PW and PW+GB represent the NLCs formulated with propolis wax alone or its binary mixture with glyceryl behenate (1:1), respectively.

In our study, the release behavior of the NLCs was performed in simulated gastric (pH 1.2) and intestine media (pH 7.4) with or without a pancreatic lipase, in comparison with a free drug dispersion (Figure 7). As can be seen, both formulations presented a very low initial β-sitosterol release in the simulated gastric fluid (about 3%), followed by a slow and sustained release (about 7%) which ultimately increased to 17% and 21% in the PW and PW+GB NLCs, respectively, over the next 18 h of incubation at the free enzyme intestine media. Such a type of drug release pattern in NLCs is most likely related to the manner of oil distribution and may indicate that the liquid lipid is incorporated homogeneously within the solid lipid matrix. The remaining high amounts of oil at the outer layers of the nanoparticles, instead of being entrapped in the solid lipid core, would lead to the formation of a soft shell with a considerable solubilization capacity towards hydrophobic drugs, which imparts the initial burst release [42,43].

In the current study, nanoparticles containing only PW as a solid lipid showed a slower drug release, probably due to the higher lipophilicity of the matrix and the presence of resins in the wax’s composition that prevents the penetration of water into the pores of the lipid structure, resulting in less hydrolysis reaction [40].

As expected, the lipase helped the digestion of the lipids in the NLCs and a higher drug release was quantified after 24 h (around 24% in the PW NLC and 28% in the PW+GB NLC) (Figure 7). Similarly, Lui et al. (2010) reported a very low contribution of a stomach enzyme (pepsin) in the release of lutein, while the simulated intestinal fluid containing a lipase improved release rate. The lipase digests the triglycerides and affects the drug release by increasing the lipid matrix porosity and the mass loss [44].

In the present study, the low and gradual release of β-sitosterol from the NLCs could be justified by its highly hydrophobic nature and remarkable affinity to the complex lipid mixture. The liquid reservoirs of oil inside the solid core lead to a high tendency of the drug to remain entrapped for a longer time inside the network lattice [45]. Another reason could be the rigid solid external shell of NLCs which may be resistant to digestion and act as a barrier for the diffusion of the encapsulated drug [46]. Both NLC formulations showed significant improvement in their drug release profiles compared with the plain drug suspension, due to dissolution enhancement [47].

In Lacatusu et al.’s (2012) study, the release of β-sitosterol from NLCs began after 4 h and reached 25% for a grape seed oil (GSO)-loaded NLC and 35% for a squalene-loaded NLC (Sq), after 24 h of release, which is comparable with the current results. The difference in the release profiles of the GSO and Sq NLCs was attributed to differences in the degree of crystallinity of the lipid matrix and the manner of the drug incorporation. In comparison with a shell-enriched model, a core-enriched pattern provides a more sustained and prolonged release [45].

### 3.4. In Vivo Study

Plant sterols are well known for their cholesterol lowering effect and can inhibit cholesterol absorption within the intestinal lumen by reducing the solubility of cholesterol and excluding it from the mixed micelles. Due to higher hydrophobicity, phytosterols are more readily absorbed into the micelles than cholesterol, leading to the fecal exertion of cholesterol. However, some studies have shown that phytosterols can be effective in reducing cholesterol even when they are not taken simultaneously with the meal, which suggests other mechanisms of action for their hypocholesterolemic activity rather than the replacement of cholesterol from the mixed micelles [3,48]. The interference with the activity of the cholesterol transporters or the incorporation of cholesterol into chylomicrons are described as other possible mechanisms [3,49].

Table 2 represents the serum lipid profiles of HCD-fed mice with respect to the control and impact of the intake of either the PW+GB formulation or free drug suspension in reducing the plasma cholesterol level. The PW+GB NLC was selected based on its higher physical stability upon storage and digestion, as well as its improved drug release profile.

Feeding on a diet enriched with cholesterol for 8 weeks caused a significant increase (*p* < 0.05) in serum total and LDL cholesterol levels, indicating the occurrence of hypercholesterolemia with HCD. Although, the free formulation of β-sitosterol showed a lower TC (*p* > 0.05) and LDL (*p* < 0.05) level compared with the HCD-fed mice, the hypolipidemic activity was significantly improved using the NLC formulation. Compared with group II, the β-sitosterol NLC-treated groups showed a marked decrease (*p* < 0.01) in TC (from 178.6 ± 7.2 to 123.4 ± 5.2 mg/dL) and LDL levels from 103.9 ±4.8 to 52.7±3.3 mg/dL) at the end of treatment (8 weeks). This could be attributed to the improved solubility and dissolution of the drug formulated as an NLC. Previous studies have shown that plant sterols are extremely effective in inhibiting cholesterol absorption when introduced into the small intestine in micellar form [49]. On the other hand, the high β-sitosterol and punicic acid content of the pomegranate seed oil may play a role in the increased hypolipidemic effect of the NLC.

Based on previous investigations, plant sterols lower the LDL cholesterol level, with no impact on the HDL cholesterol or triglyceride concentrations [3,6]. Further, in the present study, HDL cholesterol concentrations did not differ between treatments, while TG levels were found to be higher in the HCD-treated groups compared with the controls, which could be attributed to the method used for inducing the hypercholesterolemia, in line with other studies [17].

The LDL cholesterol concentrations did not change between 1 and 2 months of consumption of the plant sterols. According to previous studies, dietary phytosterols decrease the plasma cholesterol concentrations within a few weeks from the initiation of the treatment and maintains the reduced levels over 12 months of continued ingestion [6].

## 4. Conclusions

Novel β-sitosterol NLC suspensions were developed using propolis wax (alone or its binary mixture with glyceryl behenate) as a solid lipid and pomegranate seed oil as a liquid lipid. Several in vitro characterizations as well as the ability of the NLC to enhance the oral delivery and hypocholesterol activity of β-sitosterol were evaluated. The NLC dispersions exhibited relatively good physical and chemical stability during up to one-month of storage at 4, 25, and 40 °C, showing no prominent changes in the particle size, PDI, and zeta potential and only a slight decrease (2–10%) in the β-sitosterol content. The oxidative stability was appreciably better at lower storage temperatures.

During the digestion, NLCs remained almost physically stable and in the colloidal nanometer range (≤550 nm). A high β-sitosterol bioaccessibility (69% and 73% in PW and PW+GB, respectively) was observed after full digestion, due to the presence of triacylglycerol that could form mixed micelles to solubilize the hydrophobic component. The in vitro release study exhibited a gradual and sustained release of β-sitosterol, reaching around 17% and 21% in the PW and PW+GB NLCs, respectively, after 24 h, which improved in the presence of a pancreatic lipase. When tested in vivo, mice fed on the β-sitosterol NLC presented lower TC (one-half) and LDL cholesterol (two-thirds) levels as compared with the control, and an improved hypolipidemic activity in comparison with the group treated with the free β-sitosterol suspension. The TG increased in all groups receiving the high-cholesterol diet, while HDL cholesterol remained unaffected. Overall, NLCs might provide efficient nanodevices for the management of hypercholesterolemia and promising drug delivery systems to enhance β-sitosterol’s oral bioaccessibility.

## Figures and Tables

**Table 1:** Changes of antiradical activity (%) of β-sitosterol NLC dispersions during storage at different temperatures (4, 25 and 40 °C).

NLC Formulation	Temperature (°C)	Day 0	Day 10	Day 20	Day 30
PW	4	34.75 ± 0.94	34.21 ± 0.88 ^a^	34.05 ± 0.14 ^a^	33.5 ± 0.55 ^a^
PW	25	34.75 ± 0.94	34.66 ± 1.36 ^a^	34.2 ± 0.2 ^a^	33.9 ± 0.81 ^a^
PW	40	34.75 ± 0.94	34.54 ± 0.88 ^a^	34.01 ± 1.2 ^a^	33.8 ± 0.51 ^a^
PW+GB	4	31.83 ± 0.24	31.5 ± 0.71 ^b^	30.53 ± 0.57 ^b^	30.50 ± 0.13 ^b^
PW+GB	25	31.83 ± 0.24	30.33 ± 0.35 ^b^	30.2 ± 0.23 ^b^	29.8 ± 1.2 ^bc^
PW+GB	40	31.83 ± 0.24	29.96 ± 0.18 ^b^	29.7 ± 0.65 ^b^	28.6 ± 0.79 ^c^

Different a, b, c, ... letters in the same column indicate a statistically significant difference (*p* < 0.05). The means without superscript are not significantly different (*p* > 0.05).

**Table 2:** Changes in the serum lipid profile of mice after different dietary treatments.

	**Total Cholesterol (mg/dL)**		**LDL Cholesterol (mg/dL)**		**HDL Cholesterol (mg/dL)**		**Triglycerides (mg/dL)**	
**Group**	**Day 30**	**Day 60**	**Day 30**	**Day 60**	**Day 30**	**Day 60**	**Day 30**	**Day 60**
Ι	105.0 ± 5.0	107.4 ± 4.2	42.8 ± 6.7	45.9 ± 5.6	50.8 ± 8.5	52.7 ± 4.4	61.2 ± 6.7	71.3 ± 10.2
ΙΙ	137.0 ± 2.8 **	178.6 ± 7.2 **	80.4 ± 5.4 **	103.9 ± 4.8 **	42.8 ± 6.4	50.4 ± 5.6	109.4 ± 12.1 *	125.3 ± 6.0 **
ΙΙΙ	124.2 ± 4.4	153.2 ± 6.2	62.9 ± 1.3 ^	65.9 ± 3.2 ^	44.6 ± 4.9	44.6 ± 2.9	110.0 ± 9.6 *	128.6 ± 8.5 **
ΙV	117.3 ± 5.5 ^^	123.4 ± 5.2 ^^#	51.4 ± 3.2 ^^#	52.7 ± 3.3 ^^#	51.4 ± 3.3	53.4 ± 5.9	108.9 ± 110.6 *	124.9 ± 7.4 **

Group I (control group) received a normal laboratory diet (ND) and a vehicle (1% carboxymethylcellulose); group II received a high-cholesterol diet (HCD: ND supplemented with 2% cholesterol) and a vehicle. Groups III and IV were given a HCD and 40 mg/kg/day β-sitosterol as a β-sitosterol suspension in 1% carboxymethylcellulose and β-sitosterol NLC (PW+GB), respectively. ** *p* < 0.01 vs. normal diet + vehicle ^ *p* < 0.05 and ^^ *p* < 0.01 vs. HCD + vehicle # *p* < 0.05 vs. HCD + β-sitosterol suspension.

## Supplementary Materials

The following are available online at https://www.mdpi.com/1999-4923/12/4/386/s1, Table S1: Changes of the peroxide value (PV) (meq/kg oil) of the β-sitosterol NLC dispersions during storage at different temperatures (4, 25 and 40 °C).

## References

[1] Fathi H.A., Allam A., Elsabahy M., Fetih G., El-Badry M. (2018). Nanostructured lipid carriers for improved oral delivery and prolonged antihyperlipidemic effect of simvastatin. Colloids Surf. B Biointerfaces.

[2] Patch C.S., Tapsell L.C., Williams P.G., Gordon M. (2006). Plant sterols as dietary adjuvants in the reduction of cardiovascular risk: Theory and evidence. Vasc. Health Risk Manag..

[3] Brufau G., Canela M.A., Rafecas M. (2008). Phytosterols: Physiologic and metabolic aspects related to cholesterol-lowering properties. Nutr. Res..

[4] Engel R.T., Schubert H. (2005). Formulation of phytosterols in emulsions for increased dose response in functional foods. Innov. Food Sci. Emerg. Technol..

[5] Izadi Z., Nasirpour A., Garousi G., Izadi Z., Nasirpour A., Garousi G. (2012). Optimization of Phytosterols Dispersion in an Oil/Water Emulsion Using Mixture Design Approach Optimization of Phytosterols Dispersion in an Oil/Water Emulsion Using Mixture Design Approach. J. Dispers. Sci. Technol..

[6] Christiansen L.I., Lähteenmäki P.L.A., Mannelin M.R., Seppänen-Laakso T.E., Hiltunen R.V.K., Yliruusi J.K. (2001). Cholesterol-lowering effect of spreads enriched with microcrystalline plant sterols in hypercholesterolemic subjects. Eur. J. Nutr..

[7] Clifton P.M., Noakes M., Sullivan D., Erichsen N., Ross D., Annison G., Fassoulakis A., Cehun M., Nestel P. (2004). Cholesterol-lowering effects of plant sterol esters differ in milk, yoghurt, bread and cereal. Eur. J. Clin. Nutr..

[8] Lin C., Chen C., Lin Z., Fang J. (2017). Recent advances in oral delivery of drugs and bioactive natural products using solid lipid nanoparticles as the carriers. J. Food Drug Anal..

[9] Soleimanian Y., Goli S.A.H., Varshosaz J., Maestrelli F. (2018). Propolis wax nanostructured lipid carrier for delivery of β-sitosterol: Effect of formulation variables on physicochemical properties. Food Chem..

[10] Soleimanian Y., Goli S.A.H., Varshosaz J., Maestrelli F. (2019). β-sitosterol Lipid Nano Carrier Based on Propolis Wax and Pomegranate Seed Oil: Effect of Thermal Processing, pH, and Ionic Strength on Stability and Structure. Eur. J. Lipid Sci. Technol..

[11] Shantha N.C., Decker A.E. (1994). Rapid, sensitive, Iron based spectrophotometric methods for determination of peroxide values of food lipids. J. AOAC Int..

[12] Soleimanian Y., Goli S.A.H., Varshosaz J., Sahafi S.M. (2018). Formulation and characterization of novel nanostructured lipid carriers made from beeswax, propolis wax and pomegranate seed oil. Food Chem..

[13] Qiu C., Zhao M., Decker E.A., McClements D.J. (2015). Influence of anionic dietary fibers (xanthan gum and pectin) on oxidative stability and lipid digestibility of wheat protein-stabilized fish oil-in-water emulsion. Food Res. Int..

[14] Haider J., Majeed H., Williams P.A., Safdar W., Zhong F. (2017). Formation of chitosan nanoparticles to encapsulate krill oil (Euphausia superba) for application as a dietary supplement. Food Hydrocoll..

[15] Yang Y., McClements D.J. (2013). Vitamin E bioaccessibility: Influence of carrier oil type on digestion and release of emulsified α-tocopherol acetate. Food Chem..

[16] Sarkar A., Goh K.K.T., Singh H. (2009). Colloidal stability and interactions of milk-protein-stabilized emulsions in an artificial saliva. Food Hydrocoll..

[17] Katsarou A.I., Kaliora A.C., Chiou A., Kalogeropoulos N., Papalois A., Agrogiannis G., Andrikopoulos N.K. (2016). Amelioration of oxidative and inflammatory status in hearts of cholesterol-fed rats supplemented with oils or oil-products with extra virgin olive oil components. Eur. J. Nutr..

[18] McGrath J.C., Lilley E. (2015). Implementing guidelines on reporting research using animals (ARRIVE etc.): New requirements for publication in BJP. Br. J. Pharmacol..

[19] Acosta E. (2009). Bioavailability of nanoparticles in nutrient and nutraceutical delivery. Curr. Opin. Colloid Interface Sci..

[20] Charoen R., Jangchud A., Jangchud K., Harnsilawat T., Naivikul O., Mcclements D.J. (2011). Influence of biopolymer emulsifier type on formation and stability of rice bran oil-in-water emulsions: Whey protein, gum arabic, and modified starch. J. Food Sci..

[21] Mehrad B., Ravanfar R., Licker J., Joe M. (2018). Enhancing the physicochemical stability of β-carotene solid lipid nanoparticle (SLNP) using whey protein isolate. Food Res. Int..

[22] Yang Y., Leser M.E., Sher A.A., McClements D.J. (2013). Formation and stability of emulsions using a natural small molecule surfactant: Quillaja saponin (Q-Naturale). Food Hydrocoll..

[23] Khalid N., Kobayashi I., Neves M.A., Uemura K., Nakajima M., Nabetani H. (2017). Encapsulation of β-sitosterol plus γ-oryzanol in O/W emulsions: Formulation characteristics and stability evaluation with microchannel emulsification. Food Bioprod. Process..

[24] Chen X.W., Guo J., Wang J.M., Yin S.W., Yang X.Q. (2016). Controlled volatile release of structured emulsions based on phytosterols crystallization. Food Hydrocoll..

[25] Zychowski L.M., Logan A., Augustin M.A., Kelly A.L., O’Mahony J.A., Conn C.E., Auty M.A.E. (2018). Phytosterol crystallisation within bulk and dispersed triacylglycerol matrices as influenced by oil droplet size and low molecular weight surfactant addition. Food Chem..

[26] Cercaci L., Rodriguez-Estrada M.T., Lercker G., Decker E.A. (2007). Phytosterol oxidation in oil-in-water emulsions and bulk oil. Food Chem..

[27] Pool H., Quintanar D., de Figueroa J.D., Bechara J.E.H., McClements D.J., Mendoza S. (2012). Polymeric Nanoparticles as Oral Delivery Systems for Encapsulation and Release of Polyphenolic Compounds: Impact on Quercetin Antioxidant Activity & Bioaccessibility. Food Biophys..

[28] Gray D.A., Payne G., McClements D.J., Decker E.A., Lad M. (2010). Oxidative stability of Echium plantagineum seed oil bodies. Eur. J. Lipid Sci. Technol..

[29] Nasrabadi M.N., Amir S., Goli H., Nasirpour A. (2016). Stability assessment of conjugated linoleic acid (CLA) oil-in-water beverage emulsion formulated with acacia and xanthan gums. Food Chem..

[30] Comunian T.A., Ravanfar R., de Castro I.A., Dando R., Favaro-Trindade C.S., Abbaspourrad A. (2017). Combination of microfluidic devices, ionic gelation and phenolic compounds to improve the oxidative stability of echium oil. Food Chem..

[31] Guillén M.D., Cabo N. (1999). Usefulness of the frequency data of the Fourier transform infrared spectra to evaluate the degree of oxidation of edible oils. J. Agric. Food Chem..

[32] Li Y., Mcclements D.J. (2010). New mathematical model for interpreting ph-stat digestion profiles: Impact of lipid droplet characteristics on in vitro digestibility. J. Agric. Food Chem..

[33] Ozturk B., Argin S., Ozilgen M., McClements D.J. (2015). Nanoemulsion delivery systems for oil-soluble vitamins: Influence of carrier oil type on lipid digestion and vitamin D_3_ bioaccessibility. Food Chem..

[34] Aditya N.P., Shim M., Lee I., Lee Y., Im M., Ko S. (2013). Curcumin and Genistein Coloaded Nanostructured Lipid Carriers: In Vitro Digestion and Antiprostate Cancer Activity. J. Agric. Food Chem..

[35] AAditya N.P., Macedo A.S., Doktorovova S., Souto E.B., Kim S., Chang P.S., Ko S. (2014). Development and evaluation of lipid nanocarriers for quercetin delivery: A comparative study of solid lipid nanoparticles (SLN), nanostructured lipid carriers (NLC), and lipid nanoemulsions (LNE). LWT Food Sci. Technol..

[36] Van Aken G.A., Bomhof E., Zoet F.D., Verbeek M., Oosterveld A. (2011). Food Hydrocolloids Differences in in vitro gastric behaviour between homogenized milk and emulsions stabilised by Tween 80, whey protein, or whey protein and caseinate. Food Hydrocoll..

[37] Qian C., Decker E.A., Xiao H., McClements D.J. (2012). Nanoemulsion delivery systems: Influence of carrier oil on β-carotene bioaccessibility. Food Chem..

[38] Pool H., Mendoza S., Xiao H., McClements D.J. (2013). Encapsulation and release of hydrophobic bioactive components in nanoemulsion-based delivery systems: Impact of physical form on quercetin bioaccessibility. Food Funct..

[39] Fachinetti N., Rigon R.B., Eloy J.O., Sato M.R., dos Santos K.C., Chorilli M. (2018). Comparative Study of Glyceryl Behenate or Polyoxyethylene 40 Stearate-Based Lipid Carriers for Trans-Resveratrol Delivery: Development, Characterization and Evaluation of the In Vitro Tyrosinase Inhibition. AAPS PharmSciTech.

[40] Kheradmandnia S., Vasheghani-Farahani E., Nosrati M., Atyabi F. (2010). Preparation and characterization of ketoprofen-loaded solid lipid nanoparticles made from beeswax and carnauba wax. Nanomed. Nanotechnol. Biol. Med..

[41] Yuan H., Wang L.L., Du Y.Z., You J., Hu F.Q., Zeng S. (2007). Preparation and characteristics of nanostructured lipid carriers for control-releasing progesterone by melt-emulsification. Colloids Surf. B Biointerfaces.

[42] Shah N.V., Seth A.K., Balaraman R., Aundhia C.J., Maheshwari R.A., Parmar G.R. (2016). Nanostructured lipid carriers for oral bioavailability enhancement of raloxifene: Design and in vivo study. J. Adv. Res..

[43] Jia L., Zhang D., Li Z., Duan C., Wang Y., Feng F., Wang F., Liu Y., Zhang Q. (2010). Nanostructured lipid carriers for parenteral delivery of silybin: Biodistribution and pharmacokinetic studies. Colloids Surf. B Biointerfaces.

[44] Liu C., Wu C. (2010). Optimization of nanostructured lipid carriers for lutein delivery. Colloids Surf. A Physicochem. Eng. Asp..

[45] Lacatusu I., Badea N., Stan R., Meghea A. (2012). Novel bio-active lipid nanocarriers for the stabilization and sustained release of sitosterol. Nanotechnology.

[46] Kiani A., Fathi M., Ghasemi S.M. (2017). Production of novel vitamin D3 loaded lipid nanocapsules for milk fortification. Int. J. Food Prop..

[47] Elmowafy M., Ibrahim H.M., Ahmed M.A., Shalaby K., Salama A., Hefesha H. (2017). Atorvastatin-loaded nanostructured lipid carriers (NLCs): Strategy to overcome oral delivery drawbacks. Drug Deliv..

[48] Katan M.B., Grundy S.M., Jones P., Law M., Miettinen T., Paoletti R. (2003). Efficacy and safety of plant stanols and sterols in the management of blood cholesterol levels. Mayo Clin. Proc..

[49] Lees A.N.N.M., Mok H.Y.I., Lees R.S., Mccluskey M.A., Grundy S.M. (1977). Plant sterols as cholesterol-lowering agents: Clinical trials in patients with hypercholesterolemia and studies of sterol balance. Atherosclerosis.

