# Supplementary material for: β-Sitosterol Loaded Nanostructured Lipid Carrier: Physical and Oxidative Stability, In Vitro Simulated Digestion and Hypocholesterolemic Activity
Source: Pharmaceutics. 2020 Apr 22;12(4):386. doi: 10.3390/pharmaceutics12040386 (PMC7237988; doi:10.3390/pharmaceutics12040386)
Supplement: Supplementary file 1 [file pharmaceutics-12-00386-s001.pdf]

pharmaceutics

# Supplementary Materials: β-Sitosterol Loaded Nanostructured Lipid Carrier: Physical and Oxidative Stability, In Vitro Simulated Digestion and Hypocholesterolemic Activity

**Yasamin Soleimanian, Sayed Amir Hossein Goli, Jaleh Varshosaz, Lorenzo Di Cesare Mannelli, Carla Ghelardini, Marzia Cirri and Francesca Maestrelli**

**Table S1.** Changes of peroxide value (PV) (meq/kg oil) of β-sitosterol NLC dispersions during storage at different temperatures (4, 25 and 40 °C).

| NLC Formulation | Temperature (°C) | Day 0 | Day 10 | Day 20 | Day 30 |
|---|---|---|---|---|---|
| PW | 4 | 3.09±0.43 | 3.57±0.14$^{b}$ | 4.25±0.52$^{b}$ | 5.32±1.93$^{b}$ |
| PW | 25 | 3.09±0.43C | 3.99±0.34$^{b}$C | 5.9±0.61$^{ab}$B | 9.37±0.86$^{a}$A |
| PW | 40 | 3.09±0.43B | 4.87±0.86 $^{ab}$AB | 6.26±1.66 $^{ab}$A | 4.63±0.79 $^{b}$AB |
| PW+GB | 4 | 3.51±0.92 | 3.54±0.14 $^{b}$ | 4.3±0.15 $^{b}$ | 5.97±1.8 $^{b}$ |
| PW+GB | 25 | 3.51±0.92 | 4.21±0.67 $^{ab}$ | 5.57±0.37 $^{ab}$ | 3.58±0.89 $^{b}$ |
| PW+GB | 40 | 3.51±0.92B | 5.93±1.28$^{a}$AB | 7.5±0.71$^{a}$A | 5.05±1.09$^{b}$ B |

Different a, b, c, ... letters in the same column and different A, B, C, ...letters in the same row indicate a statistically significant difference ($p < 0.05$).
